# A Comparative Study on Cyanine Dyestuffs as Sensor Candidates for Macromolecular Crowding In Vitro and In Vivo

**DOI:** 10.3390/bios13070720

**Published:** 2023-07-08

**Authors:** Leon Koch, Roland Pollak, Simon Ebbinghaus, Klaus Huber

**Affiliations:** 1 Physical Chemistry, Department of Chemistry, Paderborn University, 33098 Paderborn, Germany; leonkoch@mail.upb.de; 2 Physical and Theoretical Chemistry, Faculty of Live Science, TU Braunschweig, 38106 Braunschweig, Germany; r.pollak@tu-braunschweig.de

**Keywords:** cyanine dyestuffs, sensor, macromolecular crowding, self-assembly, fluorescence, confocal microscopy, HeLa cells

## Abstract

Pseudo isocyanine chloride (PIC) has been identified in a preceding work as a sensor suited to probe macromolecular crowding both in test tubes with solutions of synthetic crowding agents and in HeLa cells as a representative of living systems. The sensing is based on a delicate response of the self-assembly pattern of PIC towards a variation in macromolecular crowding. Based on a suitable selection of criteria established in the present study, four additional cyanine dyestuffs (TDBC, S071, S2275, and PCYN) were scrutinized for their ability to act as such a sensor, and the results were compared with the corresponding performance of PIC. UV-VIS and fluorescence spectroscopy were applied to investigate the photo-physical properties of the four candidates and, if possible, light scattering was used to characterize the self-assembly of the dyestuffs in solution. Finally, HeLa cells were exposed to solutions of the most promising candidates in order to analyze their ability to infiltrate the cells and to self-assemble therein. None of the dyestuff candidates turned out to be as similarly promising in probing crowding effects in cells as PIC turned out to be. S0271 and S2275 are at least stable enough and meet the photophysical requirements necessary to act as sensors responding to changes in macromolecular crowding.

## 1. Introduction

Three types of processes are prevalent in living cells. Homogeneous catalysis by enzymes, folding and unfolding of proteins, and structure formation via self-assembly of proteins. All these processes take place in the presence of an aqueous environment with approximately 30–40% of solid content. An important part of this solid content is made up of a large variety of biological macromolecules like proteins, which due to their geometrical extension produce an excluded volume effect on all cellular components and affect cellular processes to an extent depending on the strength of the excluded effect. Excluded volume lowers the free volume available to the participants of the respective reaction, thereby increasing the activity of the reactants [1,2]. However, the significance of this excluded volume effect for any cellular process will decrease with the decreasing size of the participating components. As Minton has stated, “On geometric grounds one would not expect crowding by large macromolecules to greatly affect the behavior of small molecules or significantly smaller macromolecules, which can more easily fit into interstices between large molecules” [3].

Molecular sensors built of fluorescence dyes are widely used to monitor variations in cellular conditions such as variations in ion concentration [4], metabolites [5], or macromolecular crowding [6]. The change in the photophysical properties of dyes due to environmental changes can be directly utilized as a read-out. Alternatively, chimera sensors can be constructed from responsive biomacromolecules undergoing conformational transitions upon selective binding to the probe molecule in combination with fluorescent tags reporting probe binding.

The self-assembly of biomolecules makes up an important type of cellular process. Molecular sensors that resolve cellular conditions under which biomolecules are particularly prone to aggregate are highly desirable since such sensors could lead to a novel understanding of the disease-related mechanisms in amyloid pathologies. Chimera proteins fusing amyloidogenic and fluorescent properties were used to monitor aggregation in cells with high spatiotemporal resolution [7,8,9,10]. Chemical dyes like Thioflavin T (ThT), a benzothiazole salt, were used to resolve aggregation kinetics in vitro by monitoring the enhancement of the fluorescence due to its binding to amyloid fibrils [11].

In a different approach, simpler and more rigorously characterized systems that encompass only the dye itself can be used to investigate environmental and cellular conditions that drive self-assembly, such as a variation in mitochondrial membrane potential [12] or an increase in macromolecular crowding [13]. Both case studies rely on cyanine dyes, which are chemically similar to benzothiazoles and are fluorescent dye molecules for which aggregation is accompanied by a characteristic change in optical properties. The first of these examples used already as a sensor in cellular systems is JC-1. Environmentally dependent aggregation of that dyestuff is used to report on heterogeneities of mitochondrial membrane potential in living cells [12]. The other example, which probes macromolecular crowding in cells, is pseudo isocyanine chloride (PIC) [13]. PIC has been intensively investigated with a focus on its aggregation mechanism, aggregate structure, and its optical properties [14,15,16]. The self-assembly of PIC molecules changes their optical and photophysical properties. Unlike monomeric dye molecules, one specific type of PIC aggregate has a strong and narrow absorption maximum at 573 nm shifted towards lower energies, and exhibits fluorescence at 577 nm [17]. These aggregates are named after their discoverers Scheibe [17] and Jelley [18,19] and are called Scheibe aggregates or J-aggregates. The aggregation of PIC is temperature- and concentration-dependent, depictable as characteristic phase diagrams. The formation of J-aggregates is a reversible polymerization [20]. As this J-aggregation of PIC leads to fibrillar structures similar to various proteins, PIC turned out to be a particularly promising candidate as a protein substitute and thus as a sensor. 

PIC is cell-permeable, biocompatible, and is sensitive to aggregation in cellular environments, e.g., in *E. coli*, HeLa cells, or the nematode C. elegans. Thus, it is useful as a sensor to investigate the effect of different cellular conditions, e.g., the effect of macromolecular crowding on self-assembly processes in cells [13,15]. Taken together, these features make PIC a suitable candidate to probe environmental changes in vitro and in vivo which have an impact on the aggregation threshold [21,22].

However, the detection of the fluorescence signal of aggregated PIC in biological systems is limited by the penetration depth of the laser wavelength used for excitation, as well as by the fluorescence light emitted by the PIC [23]. Bashkatov et al. [23] investigated the penetration depth in relation to the laser wavelength and showed that light with higher wavelengths up to the near-infrared regime has the highest optical penetration depth. The excitation wavelength used for PIC is 530 nm [13] and probes tissue up to a depth of ~1 mm [23], which is a major drawback for investigating biological processes in the deeper cellular systems of living organisms. Using dyes with fluorescence peaks shifted towards higher wavelengths than PIC allows for the examination of tissue lying deeper under the surface, up to a depth of 3.5 mm [23]. Aside from J-aggregates, PIC also forms H-aggregates, complicating the interpretation of the UV-VIS absorption behavior as an additional H-shoulder appears at 482 nm. In addition, a possible monomer fluorescence differing from that of the aggregate is useful as it may allow probing of different cellular regions using the fluorescence signal of the monomer and the red-shifted fluorescence signal of the corresponding aggregate simultaneously. These desirable features that PIC lack motivated us to scan a group of promising cyanine dyes for their suitability to act as sensors with similar or even superior efficacy to PIC. 

As we have included the effect of macromolecular crowding as a condition to be probed with regard to its impact on self-assembly, the size of the sensor in relation to the size of the crowders has to be addressed explicitly. Our sensor candidates do not meet the criteria of the definition given by Minton on crowding [3]. This definition is a valuable specification serving as an excellent guide to classify effects due to macromolecular crowding. However, no clear-cut borderline exists which separates species still to be affected by a crowder from those not being affected. Even in the case of negligibly small sensors, the volume excluded by the crowders remains effective as the volume occupied by the crowder, which increases the activity of the sensor accordingly. Although this might be a fairly small effect, it is not zero. As an example [13,15], a change in the activity of PIC by 25% may easily shift the temperature threshold where self-assembly of PIC does occur by 3–5 °C. In light of subtly balanced biological systems, such small effects may already be pivotal. Above all, PIC, which is the smallest of the sensor candidates, already has a length slightly larger than 1 nm and, much more importantly, the resulting fibers in its dispersed state clearly meet the criterium given by Minton [3].

**Figure 1:**
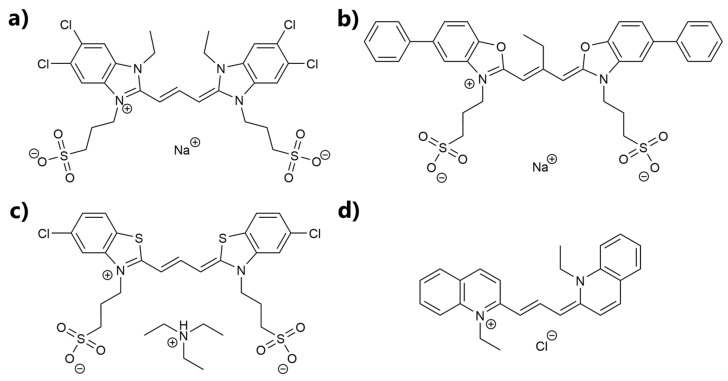
Structural formula of (**a**) TDBC, (**b**) S0271, (**c**) S2275, and (**d**) PCYN.

Accordingly, in the present work, we examined four cyanine-based dyes for possible applications as fluorescence sensors in a cellular environment. The named dyes TDBC, S0271, S2275, and Pinacyanol chloride (PCYN) are shown in Figure 1.

Following our experiences with PIC as an established sensor, the dyes in this study were examined for their sensing properties based on self-assembly. The candidates were investigated using absorption and fluorescence spectroscopy as well as light scattering. First, the UV-VIS absorption and fluorescence patterns of all four dyestuffs were examined. The formation of J-aggregates as a characteristic property of cyanine dyes was represented by means of phase diagrams based on the aggregation threshold temperature as a function of dye concentration in different electrolyte-containing solvents. Once possible, the shape and size of the J-aggregates of the dyes were successively examined using light scattering techniques in the aqueous media. Finally, the dyes were scrutinized using in vivo experiments in HeLa cells, thus providing a realistic insight for aggregation in living systems. Variable conditions were applied to learn how the cyanine dyes respond and whether these responses are suitable for application as sensors for cellular environmental changes.

The present study shall provide a direct evaluation of the performance of the dye candidates as possible sensors for the impact of cellular crowding on protein self-assembly and a comparison of the results with the corresponding performance of the reference dye PIC. In addition, it may offer useful information on the behavior of those candidates as reporter molecules in related applications or on the design of new molecules for similar applications.

## 2. Materials and Methods

### 2.1. Materials

The dyes S2275 (5-Chloro-2-[3-[5-chloro-3-(4-sulfobutyl)-3H-benzothiazol-2-ylidene]-propenyl]-3-(4-sulfobutyl)-benzothiazol-3-ium hydroxide, inner salt, triethylammonium salt, 750.83 g/mol), S0271 (3,3′-bis(4-sulfobutyl)-5,5′-diphenyl-9-ethyloxacarbocyanine betaine sodium salt, 750.86 g/mol), and TDBC (5,6-Dichlori-2-[[5,6-dichloro-1-ethyl-3-(4-sulfobutyl)-benzimidazol-2-ylidene]-propenyl]-1-ethyl-3-(4-sulfobutyl)-benzimidazolium hydroxide, inner salt, sodium salt, 762.51 g/mol) were provided by FEW Chemicals (Bitterfeld-Wolfen, Germany). The dye Pinacyanol chloride (PCYN, 2,2′-Trimethinchinocyaninchlorid, 388.93 g/mol) was obtained from Sigma-Aldrich (Steinheim am Albuch, Germany). Leibovitz’s L-15 medium was provided by Thermo Fisher (Waltham, MA, USA). LS-MS Grade water from VWR International (Radnor, PA, USA) was used to prepare the solvents. All dyes and dye solutions were stored under exclusion of light.

### 2.2. Sample Preparation

Stock solutions of the dyestuffs were made by dissolving the dyes in solvents under exclusion of light while stirring. Water, 0.1 M NaCl solution, and Leibovitz’s L-15 medium were used as solvents. The concentrations of the stock solutions in water were 50 µM for S0271, 5 mM for S2275, 1 mM for TDBC, and 10 mM for PCYN. The resulting solutions were heated at 60 °C for half an hour. Solutions with lower concentrations were prepared by diluting the stock solutions at 60 °C and heating the diluted solutions at 65 °C for 20 min. Solutions in 0.1 M NaCl were made by diluting stock solutions with concentrations of 50 µM S2275 and 20 µM S0271. The resulting solutions were treated in the same way as described above for water as a solvent. For solutions in Leibovitz’s L-15 medium, stock solutions with concentrations of 50 µM S2275 and 10 µm S0271 were diluted and treated as described for solutions in water. The resulting solutions were investigated by UV-VIS experiments starting from a temperature of 65 °C.

### 2.3. UV-VIS Experiments

UV-VIS spectra were recorded using a Lambda-19 spectrometer from PerkinElmer (Waltham, MA, USA). The spectra were recorded from 400 to 700 nm with a scan speed of 120 nm min^−1^ and a slit width of 2 mm. A custom-built copper block was used as a cuvette holder, which was thermostated externally in order to enable temperature-sensitive measurements. Water, 0.1 M NaCl, and Leibovitz’s L-15 medium were used as solvents. Depending on the transmission, a demountable cuvette with a path length of 0.01 cm (Hellma Analytics, Mühlheim, Germany) or cuvettes with a path length of 1 cm (BRAND, Wertheim, Germany) were used.

### 2.4. Static and Dynamic Light Scattering

Light-scattering experiments were performed with an ALV-CGS 5000 E instrument from ALV-Laservertriebsgesellschaft (Langen, Germany) equipped with a He-Ne laser with 35 mW (Gilching, Germany) and a wavelength of 632.8 nm. All scattering experiments were performed at T = 25 °C. Cylindrical light-scattering cuvettes from Hellma (Mühlheim, Germany) with a diameter of 1 cm were used. Prior to a light-scattering measurement, the solutions were filtered using CHROMAFIL Xtra H PTFE (0.2 µm) syringe filters (MACHEREY-NAGEL, Düren, Germany). The solutions were equilibrated in the light-scattering device for 10 min at 25 °C. The angular range of the scattering instrument is 30° ≤ θ ≤ 150°, corresponding to a range of the momentum transfer in water of 6.8 × 10^−3^ nm^−1^ ≤ q ≤ 25 × 10^−3^ nm^−1^ with

(1) $$q=\frac{4\pi n}{\lambda_{0}}\sin\left(\frac{\theta }{2}\right)$$

In Equation (1), n is the refractive index of the solvent, θ is the scattering angle, and λ_0_ is the laser wavelength in vacuum. At each q, the scattering was recorded for 10 s. Light-scattering experiments in the present work had to be restricted to the dyestuff S0271. Water was used as the solvent.

Static light scattering provides the excess Rayleigh ratio ΔR_θ_ of the solute

(2) $$\Delta R_{\theta }=RR_{\theta ,std}\left(\frac{r_{\theta ,sol}-r_{\theta ,solv}}{r_{\theta ,std}}\right)\;$$

with RR_θ,std_ being the absolute Rayleigh ratio of toluene used as standard and with r_θ,solv_, r_θ,sol_, and r_θ,std_ being the measured scattering signals of the solvent, solution, and the standard toluene.

The data from static light scattering were evaluated using the Zimm approximation [24]

(3) $$\frac{\mathrm{Kc}}{{\Delta R}_{\theta }}=\frac{1}{M_{w}}+\frac{R_{g}^{2}q^{2}}{3M_{w}}+2A_{2}c\;$$

and the Guinier approximation [25]

(4) $$\ln\left(\frac{\mathrm{Kc}}{{\Delta R}_{\theta }}\right)=\ln\left(\frac{1}{M_{w}}\right)-\frac{R_{g}^{2}q^{2}}{3}+{\mathrm{bq}}^{4}$$

with K being the contrast factor, M_w_ being the weight-averaged molar mass, R_g_^2^ being the z-averaged squared radius of gyration, with A_2_ and c (in Equation (3)) being the second osmotic virial coefficient and the concentration of the solute in g L^−1^, and with b (in Equation (4)) being an empirical coefficient of the q^4^-term. The second virial coefficient in Equation (3) is not accessible due to an expected concentration dependency of the degree of aggregation. Fortunately, the investigated concentrations were extremely low and the effect of interparticle interactions could be neglected.

The contrast factor K in Equation (3) is given as

(5) $$K=\frac{4\pi^{2}n_{0}^{2}}{N_{A}\lambda^{4}}{\left(\frac{\mathrm{dn}}{\mathrm{dc}}\right)}^{2}$$

with n_0_ being the refractive index of the index matching bath liquid (toluene) of the goniometer, with N_A_ being Avogadro’s number, and with dn/dc being the refractive index increment of the investigated solute in solution. The following constants were used: n_0_ = 1.496, n = 1.332, dn/dc = 0.621 mL g^−1^.

Evaluation of the dynamic light scattering (DLS) measurements was performed using the cumulant analysis of the electric field-time correlation function g_1_(τ) [26].

(6) $$\ln\left(g_{1}\left(\tau \right)\right)=A-\Gamma \left(q\right)\tau +k_{2}\tau^{2}$$

The constant A describes the signal-to-noise ratio, the first cumulant Γ(q) gives the inverse relaxation time of the diffusive species, and the second cumulant k_2_ quantifies the polydispersity of the solute. From Γ(q), an apparent diffusion coefficient D_z_ can be calculated according to

(7) $$D_{z}=\frac{\Gamma \left(q\right)}{q^{2}}$$

which has to be extrapolated towards q^2^ → 0 according to Equation (8)

(8) $$D_{z}=D_{0}\;\left(1+q^{2}R_{g}^{2}C+k_{d}c\right)$$

in order to extract the z-averaged diffusion coefficient D_0_. In Equation (8), C is a dimensionless parameter depending on the shape of the scattering particles and k_d_ describes the concentration dependency of the apparent diffusion coefficient D_z_. The concentration dependency of Equation (8) is neglected for the same reason as given for the corresponding neglect in Equation (3) for the SLS data.

Using the Stokes-Einstein relation, a hydrodynamically effective radius R_h_ can be calculated from the diffusion coefficient

(9) $$R_{h}=\frac{k_{b}\cdot T}{3{\pi \eta D}_{z}}$$

with k_b_ being the Boltzmann constant, T being the temperature in Kelvin, and η being the dynamic viscosity of the solvent (0.891 mPa∙s).

Further information on the structures of the scattering particles can be extracted from a comparison of the size from SLS and DLS. Accordingly, the ratio of the radius of gyration and the hydrodynamic radius is a structure-sensitive parameter defined as

(10) $$\rho =\frac{R_{g}}{R_{h}}$$

Typical values for *ρ* are 0.77 for compact spheres, 1.55 for monodisperse linear chains, and *ρ* > 2 for thin rod-like structures [27,28,29].

### 2.5. Cell Culture and Application of PIC, S0271, and S2275

HeLa cells were cultured in growth medium (Dulbecco’s Modified Eagle’s Medium from Gibco, 10% fetal bovine serum from Sigma-Aldrich, 1% penicillin-streptomycin from Gibco) at 37 °C and 10% CO_2_ in T-25 culture flasks. At 90% confluence, cells were distributed into Fluorodishes of 10 mm diameter and incubated at 37 °C and 10% CO_2_ until next day’s use. The cells were washed twice with 200 µL Dulbecco’s Phosphate-Buffered saline (DPBS, from Gibco) and 200 µL dyestuff in Leibovitz’s solution was added to the samples at concentrations of 1, 10, 50, and 100 µM. The cells were imaged after 0 and 60 min with a confocal microscope (Olympus FV3000) using an excitation wavelength of 488 nm (PIC, S0271) and 561 nm (S2275). The detection wavelength was 510 ± 20 nm for the monomer (S0271) and 570 ± 30 nm for the aggregate (S0271), and 585 ± 15 nm and 650 ± 10 nm were used for S2275 as well as 530 ± 20 nm and 575 ± 10 nm for PIC, respectively. The data were evaluated via ImageJ 1.52a.

### 2.6. Influence of the Dyestuffs on the Viability of HeLa Cells

HeLa cells cultured in Fluorodishes were washed twice with 200 µL DPBS and dyestuff (S0217, S2275, or PIC) in Leibovitz’s L-15 solution of different concentrations (10, 50, 100 µM) was added. After 1 h incubation at 37 °C and 10% CO_2_, the samples were washed twice with 200 µL DBPS and 25 µL Trypsin (Sigma-Aldrich) was added to detach the cells from the surface. After 5 min incubation, the cell suspension was taken to a reaction tube. A total of 10 µL of the cell suspension was transferred to another reaction tube and combined with 10 µL Trypan blue (Sigma-Aldrich). After mixing, the solution was taken to a Neubauer chamber for cell counting and viability evaluation.

## 3. Results

### 3.1. Optical Spectroscopy of the Cyanine Dyes

**Figure 2:**
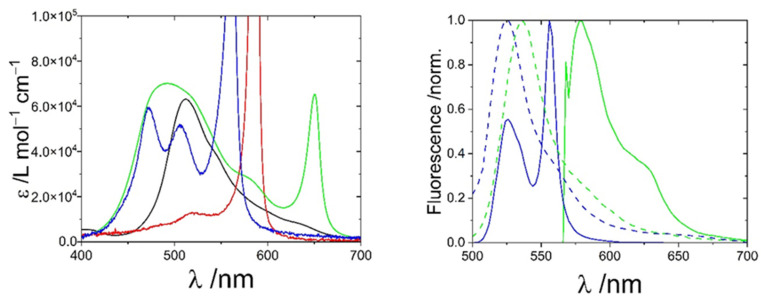
UV-VIS (**left**) and fluorescence (**right**) spectra of the dyes PCYN (black), TDBC (red), S2275 (green), and S0271 (blue) in the aggregated phase and in the monomeric state (dashed). The fluorescence spectrum of TDBC is shown in Ref. [29].

Four cyanine dyes are investigated in the present work. Absorption of the dyestuff monomers except for TDBC, which is given in Ref. [29], are summarized in Figure S1. UV-VIS spectra of these dyes in water in aggregated states are shown in Figure 2. All dyes, except for PCYN, show absorption in the range of 400–700 nm and a red-shifted, sharp absorption peak which can be assigned to J-aggregates. The wavelength of the J-peaks is shown in Table 1. PCYN does not exhibit a sharp J-peak but only a weak shoulder at around 640 nm instead.

v. Berlepsch et al. [30] showed that the formation of J-aggregates of PCYN is slow and extends over a period of several months. The generation of PCYN J-aggregates depends on the concentration as well as on the sample preparation and can be triggered by shear stress. They also observed that the formation of J-aggregates in solution prepared by dissolving the dye in solvent differs from the formation of J-aggregates in solution based on diluting stock solutions. UV-VIS experiments shown in Figure S2 confirm the slow formation process. The monomer peak of PCYN slowly decreases with time, whereby a new shoulder at 650 nm is forming most likely due to the aggregation of monomers.

Unfortunately, PCYN does not show any fluorescence in monomers or aggregates. This complete lack of fluorescence together with the extremely slow formation process of J-aggregates forced us to abstain from further consideration of PCYN in the present work.

**Table 1:** Wavelength of the maximum absorption peak λ_J_ and of the fluorescence peak λ_Jf_ of the J-aggregates, the J-aggregation threshold [dye]_JA_ at 20 °C, and the fluorescence peak of monomers λ_mf_.

	PCYN	TDBC	S0271	S2275
λ_J_ [nm]	640 ^(a)^	586	561	650
[dye]_JA_ at T_JA_ = 20 °C	-	-	1 µM	0.6 mM
λ_Jf_ [nm]	-	583 ^(b)^	556	625
λ_mf_ [nm]			525	586

(a) shoulder; (b) see Ref. [31]

The dye TDBC shows a strong absorption peak at 586 nm in its state as a J-aggregate, which is well separated from its monomer absorption spectrum (400–550 nm). Herz et al. [32] showed that TDBC has only two active species, monomers and J-aggregates. In contrast to most cyanine dyes which form J-aggregates and H-aggregates, TDBC does not show any formation of H-aggregates, which would simplify the proper interpretation of the absorption spectra. In addition, TDBC exhibits fluorescence in its aggregate as well as in its monomeric state. This special feature may allow a detailed tracking of the diffusion of dye monomers in the cellular environment to any desired location before larger and fewer mobile aggregates are formed. Unfortunately, our UV-VIS experiments with TDBC do not show sufficient stability of the aggregates on a time scale which is needed for a proper investigation of cellular processes (Figure S3). We therefore also exclude TDBC as a suitable candidate for sensing cellular environments.

The dyes S0271 and S2275 show a sharp J-peak at 561 nm and 650 nm, respectively, as well as fluorescence, and thus meet the requirements for an application in sensing cellular environments using microscopic fluorescence techniques. An alternative mode to self-assembly leads to so-called H-oligomers, which exhibit a characteristic blue shift in their absorption peaks. Both dyestuffs exhibit blue shifts in the UV-VIS at concentrations already well above the aggregation threshold, indicating the existence of H-oligomers (Figure S1) in addition to monomeric species, very much in the same way as has been observed with PIC [33]. In comparison to the well-investigated cyanine dye PIC, S2275 has its J-peak shifted to higher wavelengths, enabling studies in tissue requiring a deeper penetration depth [23]. Unfortunately, this shift of the J-peak to higher wavelengths also causes non-negligible absorption of light at the wavelength of the He-Ne laser of the light-scattering device, preventing us from performing meaningful SLS and DLS experiments with aggregated S2275. In contrast to S2275, the J-maximum of S2071 is slightly shifted to lower wavelengths compared to PIC [13,34].

Accordingly, it is S0271 and S2275 which shall be further analyzed for their suitability as sensors.

### 3.2. Threshold Temperature of Cyanine Dyes

A monomer addition process with a loss in entropy and a gain in enthalpy is observed for many chain reactions in polymer chemistry. The threshold temperature in such chain reactions, where polymerization and depolymerization are at equilibrium, is called the ceiling temperature. As mentioned, the self-assembly of PIC is comparable to such a reversible polymerization via a chain reaction, which can be promoted by decreasing the temperature [35]. Therefore, we introduce a threshold temperature T_JA_ where J-aggregates appear for the first time at a given dye concentration, in analogy to this ceiling temperature.

The appearance of the J-peak at the wavelength given in Figure 2 is the characteristic feature used to analyze the phase behavior of J-aggregates. In the present study, the self-assembly of monomeric dye will be followed by a time-dependent recording of the aggregate absorbance during a temperature gradient. 

**Figure 3:**
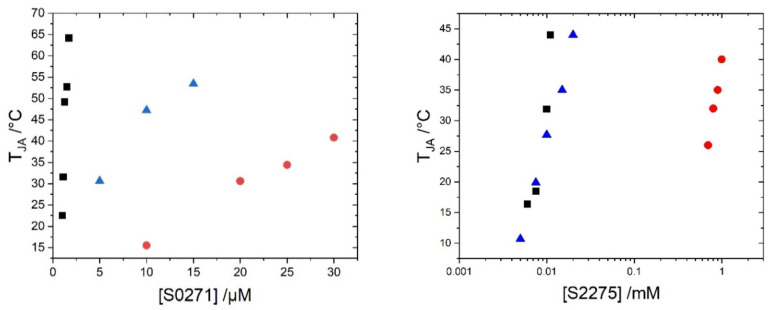
Temperature threshold of the formation of J-aggregates for S0271 and S2275 as a function of dye concentration in water (red ●), 0.1 M NaCl in water (blue ▲), and Leibovitz’s solution (black ■).

Temperature-dependent measurements for investigating the aggregation behavior reveal two regimes, a constantly low absorption at λ_J_ above and a strongly increasing absorption at λ_J_ below a threshold temperature. The low absorption regime indicates the absence of significant amounts of J-aggregates and thus temperatures above the aggregation threshold. At this threshold temperature, the absorption starts to increase with decreasing temperature, thereby establishing the second regime. The aggregation thresholds were established by linear extrapolation of the two regimes with the intersection of both extrapolations corresponding to the aggregation threshold temperature plotted in Figure 3. An example of such a fitted temperature-resolved UV-VIS measurement is given in Figure S4.

In order to provide suitable reference data for the application of those dyes as probes in biological environments, the aggregation thresholds of S0271 and S2275 were measured in three solvents including pure water, salt solution (0.1 M), and Leibovitz’s L-15 medium (Figure 3). The presence of salt in solutions of S2275 and S0271 shifts the aggregation threshold to lower concentrations. At room temperature, the concentration of dye required to reach the threshold in the case of S0271 is decreased by a factor of 1/4 by adding 0.1 M NaCl to pure water. The influence of Leibovitz’s medium on the aggregation of S0271 leads to a further shift of the threshold. This may be explained by the complex composition of Leibovitz’s medium, which aside from NaCl (138 mM) contains many amino acids and vitamins and several types of inorganic salts. In the case of dye S2275, the influence of Leibovitz’s medium is the same as of 0.1 M NaCl in water. In both media, the threshold is shifted by two orders of magnitude to lower concentrations compared to the aggregation threshold in pure water. Hence, the aggregation of S2275 is strongly influenced by salt.

### 3.3. Determination of the Size and Shape of Aggregates

The size and shape of dye aggregates were analyzed by combined SLS and DLS. As has been outlined in Section 3.1, dyestuff S2275 does not allow meaningful light-scattering experiments and therefore only solutions of dye S0271 were characterized via light scattering. The investigated concentrations are far below the aggregation threshold shown in Figure S5. The concentrations used for evaluating the molar mass of the aggregates with Equation (3) or Equation (4) are calculated by subtracting the dye concentration of the aggregation threshold from the total dye concentration, resulting in the dye concentration of aggregates.

**Figure 4:**
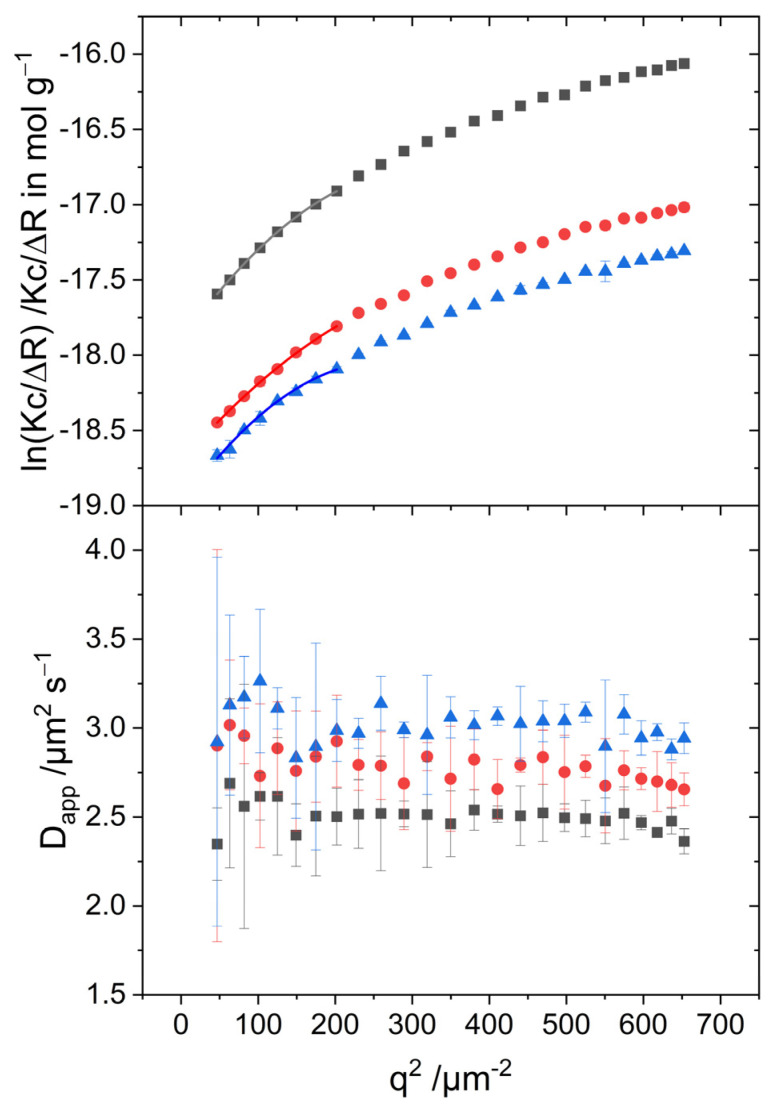
Static and dynamic light scattering of aggregated S0271 investigated for 45.7 µM (black ∎), 30.2 µM (red ●), and 27.1 µM (blue ▲) 1 h after sample preparation. The lines added to the SLS curves indicate the analysis of the respective data with the Guinier approximation given by Equation (4).

SLS and DLS experiments were carried out 1 h and 24 h after preparation of the respective solution in order to explore the self-assembly as well as the long-term stability of the aggregates. Results recorded 1 h after sample preparation are shown in Figure 4. The values for the radius of gyration and molar mass from SLS, and for the hydrodynamic radius from DLS, are summarized in Table 2. The values for the radii of gyration obtained from the Zimm approximation are slightly larger than those obtained from the Guinier approximation. Both approximations result in radii of gyration in the range of 140–160 nm. The hydrodynamic radii of the J-aggregates adopt values of 80–100 nm. The resulting ratio of R_g_/R_h_ ~ 1.7 is typical for semi-flexible chains or chains under good solvent conditions [27,28,29]. After 24 h, the radii of gyration, as well as the hydrodynamic radii, slightly increased to values of R_g_ ~ 210–230 nm and R_h_ ~ 115–130 nm, respectively, while the structure-sensitive ratio ρ remained close to ~1.6 (see Table S1 for details). The weight-averaged molar mass extracted with both approximations adopt values in the order of magnitude of 10^8^ g/mol.

**Figure 5:**
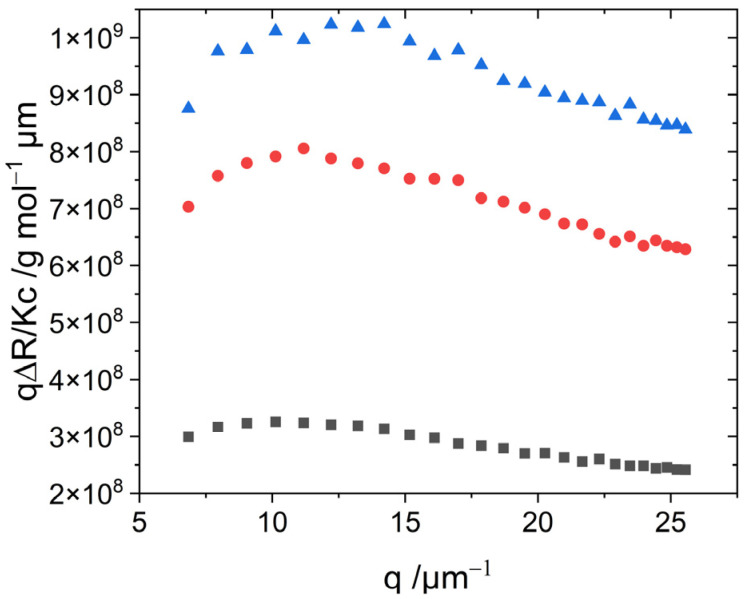
Bending-rod plot of scattering curves from solutions of aggregated S0271 investigated for 45.7 µM (black ∎), 30.2 µM (red ●), and 27.1 µM (blue ▲) 1 h after sample preparation.

A bending-rod plot of the SLS curves shown in Figure 5 reveals a maximum, which is characteristic for semi-flexible chains [36]. The curves measured 24 h after preparation (Figure S6) even approach the plateau corresponding to the q^−1^ decay of the scattering intensity as it is caused by semi-flexible or rigid rods [37]. This plateau enables calculation of the linear mass density M_L_ of the fibers [38].

(11) $$q\left(\frac{R_{\theta }}{Kc}\right)\;\;\vec{\;large\;q}\;\;\;\pi M_{L}$$

A calculation of M_L_ for those samples adopts values of M_L_ ~ 5 × 10^5^ g mol^−1^ nm^−1^ with Equation (11). Assuming a cylindrical shape of the aggregates, a cylinder volume can be calculated using M_L_, M_w_, and an estimated density of aggregates of 1 g/cm^3^. From the obtained cylinder volume, the cross-section can then be inferred, which is d = 20 nm, corresponding to a cylinder radius of r = 10 nm.

The values for the structure-sensitive ratio ρ together with the trend of the bending-rod plots suggest further analysis of the scattering curves with the model of worm-like cylinders. The fit parameters of worm-like cylinders include the contour length L, the Kuhn length b, and the radius of the cylinder cross-section r. In addition, a distribution of the contour length of the cylinders was considered via a Schulz distribution. All fits were performed with the SasView software [39]. Details of the fitting and the results thereof are summarized in Table S2 of the Supplementary Material. As fits based on a cross-section radius of 5 nm or 10 nm have almost the same quality (Table S2 of the Supplementary Material) and the mass per unit length extracted from Equation (11) is in favor of a cross-section radius of 10 nm, further discussion will be restricted to the model fits based on r = 10 nm.

It is apparent that the molar mass of the S0271 aggregates are roughly two orders of magnitude larger than the molar mass values observed for PIC aggregates in 0.01 M NaCl [16]. This discrepancy cannot be attributed only to the longer contour lengths observed with S0271. The main cause certainly lies in the larger mass per unit length M_L_ ~ 5 ×10^5^ g mol^−1^ nm^−1^ observed with S0271 as compared to M_L_ ~ 4.5 ×10^3^ g mol^−1^ nm^−1^ of PIC in 0.01 M NaCl and thus in a fiber cross-section, which is significantly larger for S0271 than for PIC. 

Form factor fits to scattering curves measured immediately after sample preparation revealed contour lengths in the range of 700–1000 nm, Kuhn lengths close to 120 nm, and cylinder cross-section radii not larger than 10 nm. Extrapolation of the form factor fits to q = 0 results in M_w_ of fitted model curves in analogy to Equation (3). As can be inferred from a comparison of the data from Table 2 with data from Table 3 and of data from Table S1 with data from Table S2, the M_w_ obtained from Zimm or Guinier approximation and from extrapolating the form factor fits are consistent.

Form factor fits to scattering curves recorded 1 h after sample preparation (Figure S6) lead to longer contour lengths, whereas chain stiffness characterized by the Kuhn length as well as the cross-section given by the cylinder radius is not affected significantly. In accordance with this, the molar mass M_w_ increases by a factor of 2–4. Hence, the aging of the J-aggregates of S0271 is manifested by the elongation of the semiflexible chains. However, as the elongation always remains below a factor of 2 but M_w_ increases typically by a factor of 3–4, elongation of the fibers hast to be accompanied by an increase of its linear mass density. 

### 3.4. Applicability of S0271 and S2275 as Self-Assembly Sensors in Living Cells in Comparison to PIC

**Figure 6:**
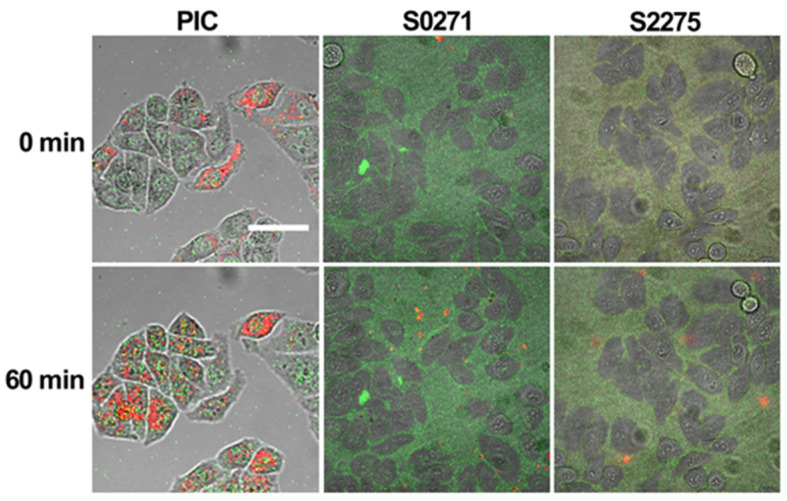
Example of HeLa cells treated with Leibovitz’s medium supplemented with 10 µM PIC (first column), 1 µM S0271 (second column), or 1 µM S2275 (third column) at t = 0 min and 60 min after application. The monomer of each dye is displayed in green and the aggregate in red. The scale bar represents 50 µm.

We tested the applicability of S0271 and S2275 as aggregation sensors in HeLa cells in comparison to PIC. Cells were treated for 1 h with Leibovitz’s medium supplemented with 1, 10, 50, and 100 µM of the respective dyes. The lowest concentration was selected to facilitate at least one experiment, where no aggregation of the dyestuff occurs in Leibovitz’s solution prior to and during infiltration. Confocal live cell imaging was used to monitor the localization and aggregation state of the dye. S0271 and S2275 immediately accumulated as monomers in the cytoplasm of HeLa cells even at the lowest concentration (1 µM) after the application of the dye (0 min) (Figure 6). After 60 min of incubation, no further accumulation was observed. However, the in-cell accumulation of the dyes was rather low. At higher concentrations (10, 50, and 100 µM), more aggregation in the cells was expected due to the possible increase in monomer concentration inside the cell. However, we found that under these conditions, the dyes mostly aggregated in Leibovitz’s medium exposed to the cells, in accordance with the phase diagrams shown in Figure 3. The aggregates were impermeable to cells and sediment onto the monolayer of cells in the cell culture, leading to an enrichment at the contact sites of neighboring cells (Figure S8), which resulted in an increased background fluorescence that impaired the imaging quality. Importantly, at higher concentrations, the cells in culture are not homogenously infiltrated by the dyes. Only up to 60% of the cells show fluorescence under any condition. In the case of infiltration, significant aggregation of S0271 or S2275 inside the cells was not observed under any condition. Furthermore, the wavelength of fluorescence for the aggregate was not very separable from that of the monomer due to spectral overlap. On the other hand, PIC was able to infiltrate 100% of the cells’ cytoplasm at each concentration (Figure 6, Figure S8) and aggregated only within the cellular environment due to crowding even when the concentration in solution was well below the aggregation threshold. Therefore, cellular crowding does not trigger aggregation, which renders the use of S0271 and S2275 as crowding sensors questionable. Finally, we tested the viability of HeLa cells treated with different concentrations of the dyes. After one hour of treatment all three dyes showed a similar minor decay in cell number, listed in Table 4 (in Figure S9 visualized as diagram), showing that in this regard the dyes are equally applicable for in cell measurements.

## 4. Conclusions

Based on its ability to form fiber-like J-aggregates and to show fluorescence in its aggregated state, PIC turned out to act as a sensor that responds to changes in crowded environments like osmotic stress, which increases macromolecular crowding in cells. These findings motivated us to select four cationic cyanine dyestuffs, PCYN, TDBC, S0271, and S2275, all of them closely related to PIC, and to examine them for their potential to respond to environmental changes in the same or in a similar way as PIC does and as self-assembling proteins would do. They have thus been selected as they all form fiber-like aggregates and were supposed to signal this feature via suitable photophysical properties. The criteria required to make those dyestuffs successful candidates are defined as follows: (i) the dyestuff has to be chemically stable both in vitro and in vivo; (ii) the dyestuff forms J-aggregates fast enough in vitro and in vivo, enabling it to respond to environmental changes in due time; (iii) the dyestuff shows a fluorescence signal in its aggregated state, as only this feature would make it a reporter molecule in cellular surroundings; (iv) the dyestuff is able to infiltrate cells. PIC has been used as a reference system fulfilling these criteria and has been used successfully in a first attempt to signal osmotic stress in cells as a potential trigger for self-assembly. Two additional features would bring any of these dyestuff candidates even beyond the quality of the reference system PIC. One feature is the lack of any H-oligomers or aggregates, which would render interpretation of the photophysical behavior less demanding than in the case of PIC. The other feature is an additional fluorescence signal from monomers differing in wavelength from that of the J-aggregate, which would enable an independent analysis of the special distribution of monomers. A detailed investigation of the four dyestuffs with respect to the criteria established above revealed the following results. 

The tendency of the dyestuff PCYN to form J-aggregates turned out to be too slow for an anticipated use as a reporter system. Most importantly, it lacks fluorescence. Both deficiencies ruled out any application in sensing environmental changes pivotal to self-assembly.

The dyestuff TDBC had to be discarded as a candidate simply due to the stability of the J-aggregates, which was insufficient for a proper investigation of cellular processes.

The two remaining dyestuffs passed the requirements posed by criteria i, ii, and iii. However, only the J-aggregates of dyestuff S0271 could be analyzed by means of significant SLS and DLS experiments as the corresponding aggregates of S2275 showed absorption in the spectral regime, in which the laser wavelength of the light-scattering instrument is located.

Remarkably, S0271 and S2275 have the ability to infiltrate HeLa cells as monomers. However, unlike PIC, neither S0271 nor S2275 showed any self-assembly in cells. Both dyestuffs were only present as monomers in the cellular environment and only rather low accumulations occurred. This may not necessarily be a drawback, because once aggregation occurred in cells, the reservoir would cause further supply of dyestuff, albeit at a low concentration. Increasing the level of dyestuff in the supernatant did not help as that triggered aggregation in the supernatant. A more serious problem may be posed by the fact that, independent of the dyestuff concentration in the supernatant, not more than up to 60% of the cells showed significant infiltration of S0271 and S2275. However, since cellular systems are highly complex systems, macromolecular crowding by far is not the only trigger of self-assembly processes in cells. Therefore, S0271 and S2275 may serve as sensor systems for other triggers for self-assembly in cells due to their excellent photophysical properties connected to their aggregation patterns. Despite these features, none of the examined dyestuffs meet the requirements for a sensor system for molecular crowding like PIC does.

## Figures and Tables

**Table 2:** Radius of gyration R_g_, hydrodynamic radius R_h_, molar mass M_w_, and the structure-sensitive parameter ρ using the Zimm and Guinier approximation for three concentrations of S0271 (27.1 µM, 30.2 µM, and 45.7 µM) measured 1 h after preparation of the solutions.

			Zimm			Guinier	
C [µM]	R_h_ [nm]	R_g_ [nm]	M_w_ [g/mol]	Ρ	R_g_ [nm]	M_w_ [g/mol]	ρ
27.1	81.2	144	1.4 × 10^8^	1.77	139	1.7 ×10^8^	1.71
30.2	87.9	156	1.6 ×10^8^	1.77	133	1.3 ×10^8^	1.51
45.7	97.7	164	6.2 ×10^7^	1.69	147	5.9 ×10^7^	1.50

**Table 3:** Contour length L, Kuhn length b, cylinder radius r, polydispersity factor PD, χ^2^, M_w_, and radius of gyration R_g_ obtained from form factor fits using SasView to scattering curves measured at S0271 concentrations for 27.1 µM, 30.2 µM, and 45.7 µM 1 h after sample preparation.

C/µM		L/nm	b/nm	r/nm	PD	Χ^2^	M_w_/g mol^−1^	R_g_/nm
27.1	1 h	716.9	123.7	10	0.4	0.90	1.2 ×10^8^	137.6
30.2	1 h	917.4	85.3	10	0.4	195.5	9.8 ×10^7^	129.2
45.7	1 h	1031.4	113.3	10	0.4	2.31	4.8 ×10^7^	157.9

**Table 4:** Count and viability of HeLa cells after 1 h treatment with dye S0271, S2275, and PIC at different concentrations in Leibovitz’s buffer.

	Reference	S0271			S2275			PIC		
[dye] /µM		10	50	100	10	50	100	10	50	100
Cell count [×10^3^]	34.0	7.5	20.0	22.0	25.5	31.5	20.6	30.7	37.4	36.0
Cells alive [%]	100	89.96	84.02	77.84	94.52	78.97	71.81	90.36	81.71	77.73

## Data Availability

Data reported in the study are available from the corresponding author on reasonable request.

## Supplementary Materials

The following supporting information can be downloaded at:https://www.mdpi.com/article/10.3390/bios13070720/s1, Figure S1. (a) UV-VIS spectra of different concentrations of S0271 (10 µM, 20 µM, 25 µM, and 30 µM) at 50 °C. (b) UV-VIS spectra of different concentrations of S2275 (0.01 mM, 0.1 mM, 0.25 mM, and 0.5 mM) at 20°C. (c) Overview of the monomer spectra from S0271 (10 µM), S2275 (10 µM), and PCYN (2 µM). All spectra have been recorded above the threshold line indicating the onset of J-aggregate formation of the respective dyestuff. The spectral changes with varying concentrations suggest the existence of small H-oligomers in addition to monomers; Figure S2. UV-VIS spectra of PCYN measured 1 day (black), 3 days (red) and 9 days (blue) after sample preparation; Figure S3. Time dependent UV-VIS absorbance of 0.06 mM TDBC solution measured at a wavelength of 586 nm. The decrease in the absorbance is due to the instability of the TDBC aggregates. No precipitation was observed during the measurement; Figure S4. Evolution of the absorbance of an S2275 solution in 0.1 M NaCl recorded at the wavelength of 650 nm with time at an S2275 concentration of 7.5 µM; Table S1. Radii of gyration Rg, hydrodynamic radii Rh, molar mass Mw, and the structure-sensitive parameter ρ for three aggregated samples (44.7 µM, 47.4 µM, and 63.3 µM) measured for freshly prepared and 24-hour-old solutions using the Zimm and Guinier approximations; Figure S5. Aggregation threshold of S0271 in water (solid line) and the investigated dye concen-trations (X) using light scattering; Figure S6. Bending-rod plot of aggregated S0271 investigated for 45.7 µM (∎, ⧠), 30.2 µM (●, o) and 27.1 µM (▲, Δ) 1 h (filled symbols) and 24 h (hollow symbols) after preparation of the dye solution; Figure S7. Static and dynamic light scattering of aggregated S0271 investigated for 45.7 µM (∎, ⧠), 30.2 µM (●, o) and 27.1 µM (▲, Δ) for 1 h (filled) and 24 h (hollow) after the preparation of the dye solutions. The static light scattering was analyzed using the Guinier and Zimm approximations; Table S2. Contour length L, Kuhn length b, cylinder radius r, polydispersity factor PD, χ^2^, Mw, and radius of gyration Rg obtained from form factor fits using SasView to scattering curves measured for S0271 solutions at concentrations of 27.1 µM, 30.2 µM, and 45.7 µM 1h and 24h after sample preparation; Figure S8. Exemplar HeLa cells treated with Leibovitz’s medium supplemented with PIC (50 and 100 µM), S0271, and S2275 (10, 50, and 100 µM) at t = 0 min and 60 min after application. The monomer of each dye is displayed in green and the aggregate in red. The scale bar represents 50 µm; Figure S9. Visualization of the viability of the cells after 1 h of dye treatment [40].
