# Supplementary material for: A Comparative Study on Cyanine Dyestuffs as Sensor Candidates for Macromolecular Crowding In Vitro and In Vivo
Source: Biosensors (Basel). 2023 Jul 8;13(7):720. doi: 10.3390/bios13070720 (PMC10377163; doi:10.3390/bios13070720)
Supplement: Supplementary file 1 [file biosensors-13-00720-s001.zip › biosensors-2384320-supplementary.pdf]

biosensors

Supporting Information

A Comparative Study on Cyanine Dyestuffs as Sensor Candidates for Macromolecular Crowding In Vitro and In Vivo

Leon Koch [1], Roland Pollak [2], Simon Ebbinghaus [2,*] and Klaus Huber [1,*]

[1] Physical Chemistry, Department of Chemistry, Paderborn University, 33098 Paderborn, Germany; leonkoch@mail.upb.de
[2] Physical and Theoretical Chemistry, Faculty of Live Science, TU Braunschweig, 38106 Braunschweig, Germany; r.pollak@tu-braunschweig.de
* Correspondence: s.ebbinghaus@tu-braunschweig.de (S.E.); klaus.huber@upb.de (K.H.)

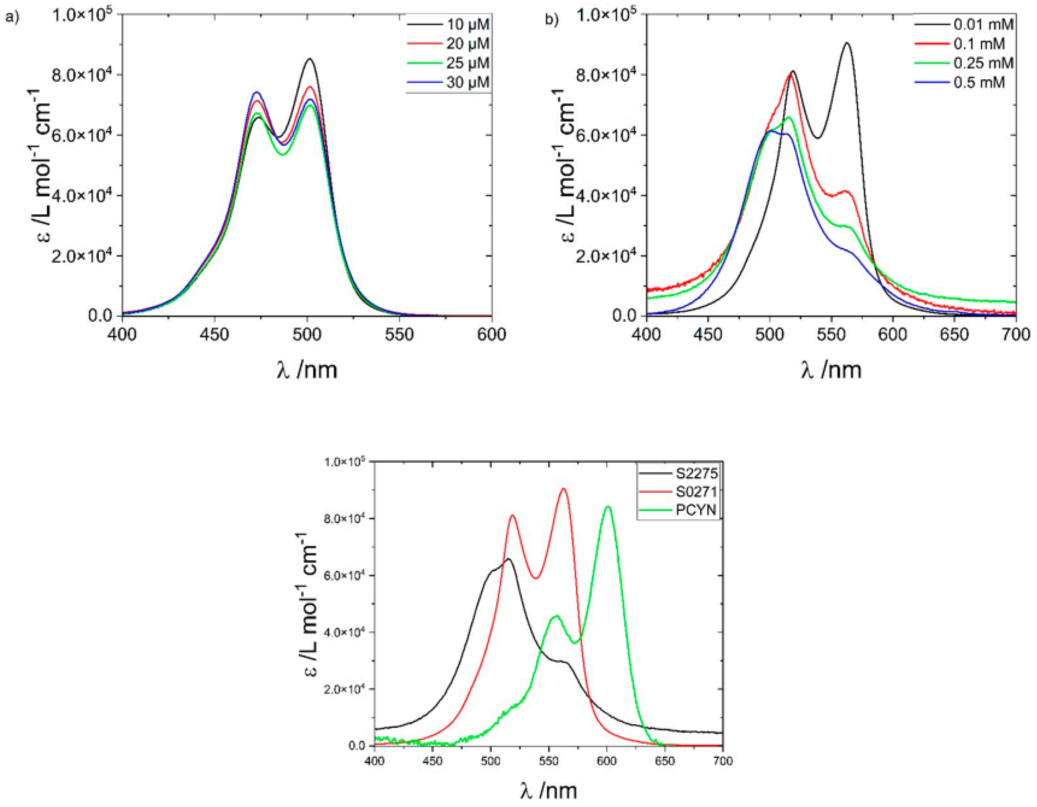

Figure S1. (a) UV-VIS spectra of different concentrations of S0271 (10 µM, 20 µM, 25 µM, and 30 µM) at 50 °C. (b) UV-VIS spectra of different concentrations of S2275 (0.01 mM, 0.1 mM, 0.25 mM, and 0.5 mM) at 20°C. (c) Overview of the monomer spectra from S0271 (10 µM), S2275 (10 µM), and PCYN (2 µM). All spectra have been recorded above the threshold line indicating the onset of J-aggregate formation of the respective dyestuff. The spectral changes with varying concentrations suggest the existence of small H-oligomers in addition to monomers.

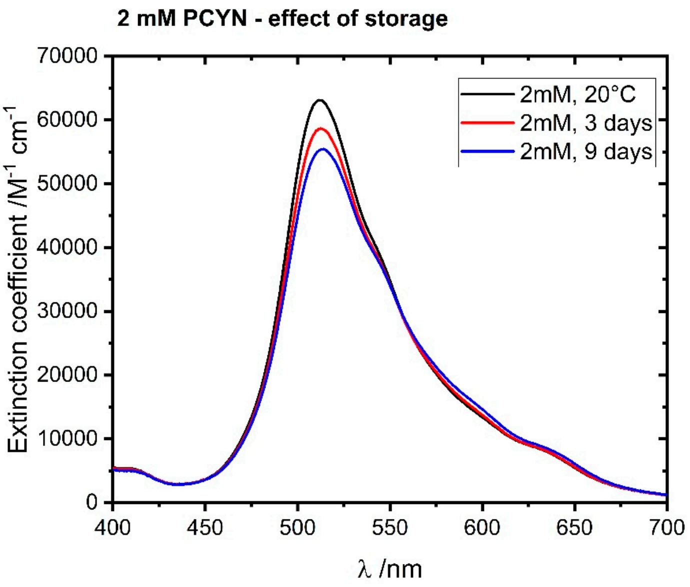

Figure S2. UV-VIS spectra of PCYN measured 1 day (black), 3 days (red) and 9 days (blue) after sample preparation.

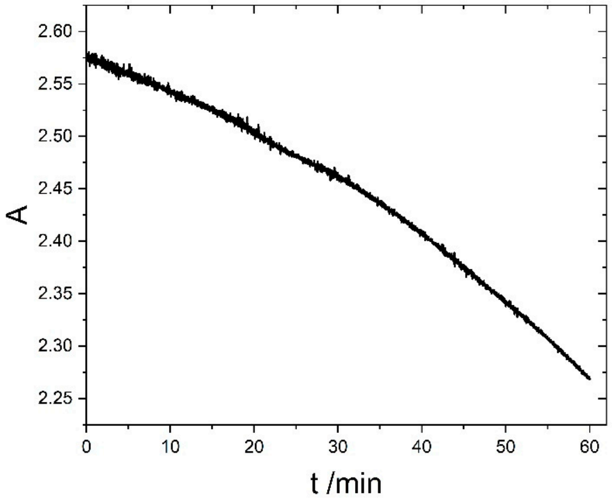

Figure S3. Time dependent UV-VIS absorbance of 0.06 mM TDBC solution measured at a wavelength of 586 nm. The decrease in the absorbance is due to the instability of the TDBC aggregates. No precipitation was observed during the measurement.

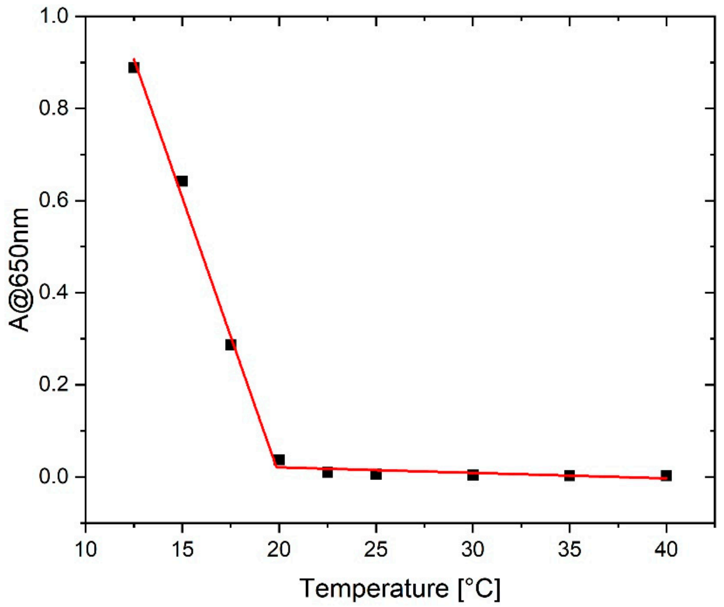

Figure S4. Evolution of the absorbance of an S2275 solution in 0.1 M NaCl recorded at the wavelength of 650 nm with time at an S2275 concentration of 7.5 µM.

Table S1. Radii of gyration Rg, hydrodynamic radii Rh, molar mass Mw, and the structure-sensitive parameter ϱ for three aggregated samples (44.7 µM, 47.4 µM, and 63.3 µM) measured for freshly prepared and 24-hour-old solutions using the Zimm and Guinier approximations.

			Zimm			Guinier	
**C [µM]**	**R_h [nm]**	**R_g [nm]**	**M_w [g/mol]**	**ϱ**	**R_g [nm]**	**M_w [g/mol]**	**ϱ**
27.1	81.2	144	$1.0 \cdot 10^8$	1.77	139	$1.0 \cdot 10^8$	1.71
30.2	87.9	156	$9.1 \cdot 10^7$	1.77	133	$8.5 \cdot 10^7$	1.51
45.7	97.7	164	$4.5 \cdot 10^7$	1.69	147	$4.3 \cdot 10^7$	1.50
27.1 24h	116.9	212	$4.3 \cdot 10^8$	1.81	178	$4.0 \cdot 10^8$	1.52
30.2 24h	130	234	$3.5 \cdot 10^8$	1.80	182	$3.0 \cdot 10^8$	1.40
45.7 24h	119	237	$1.2 \cdot 10^8$	1.99	193	$1.1 \cdot 10^8$	1.62

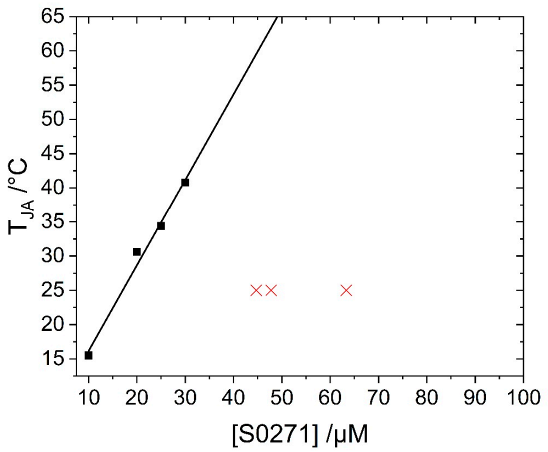

Figure S5. Aggregation threshold of S0271 in water (solid line) and the investigated dye concentrations (X) using light scattering.

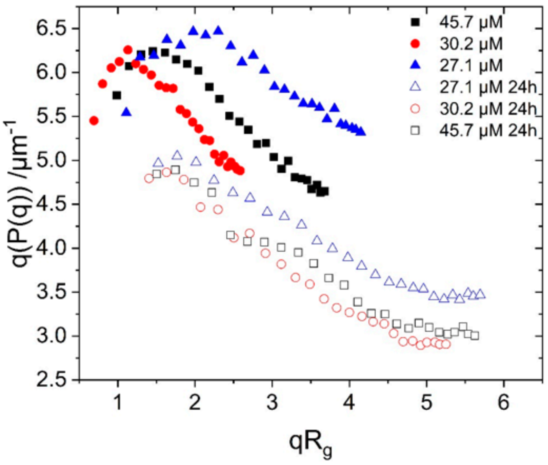

Figure S6. Bending-rod plot of aggregated S0271 investigated for 45.7 µM (■, □), 30.2 µM (●, o) and 27.1 µM (▲, Δ) 1 h (filled symbols) and 24 h (hollow symbols) after preparation of the dye solution.

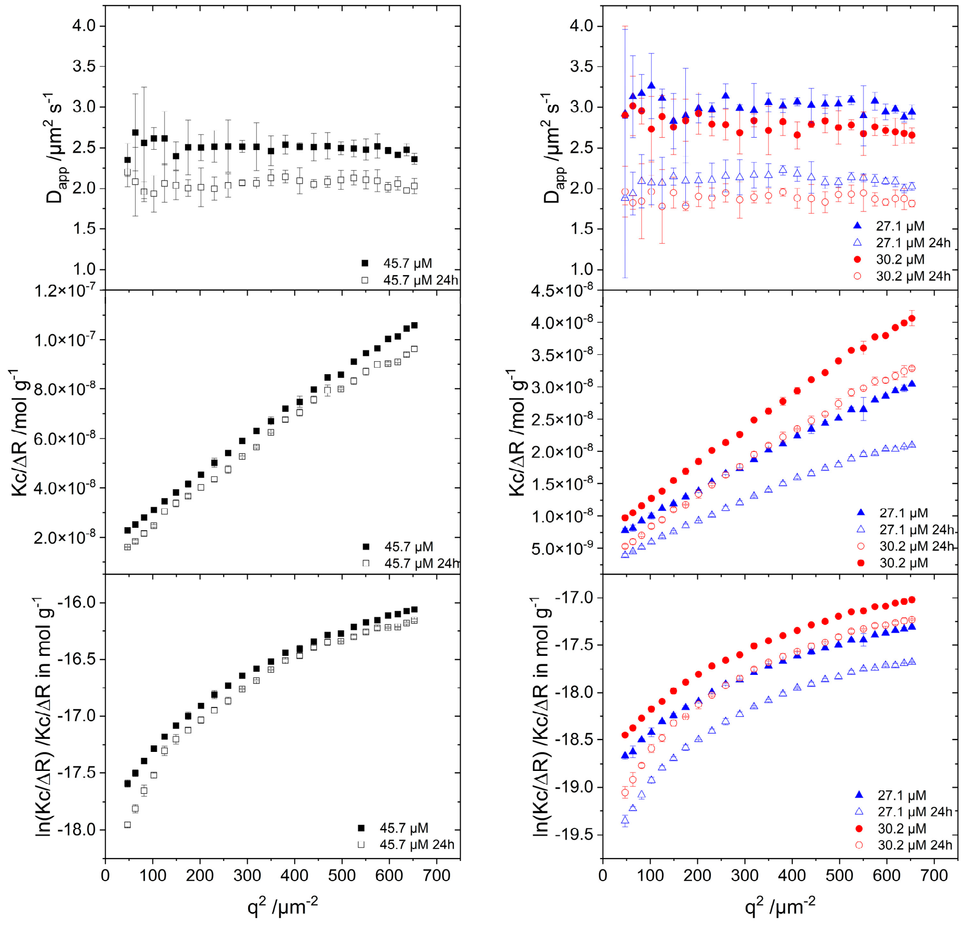

Figure S7. Static and dynamic light scattering of aggregated S0271 investigated for 45.7 µM (■, □), 30.2 µM (●, o) and 27.1 µM (▲, Δ) for 1 h (filled) and 24 h (hollow) after the preparation of the dye solutions. The static light scattering was analyzed using the Guinier and Zimm approximations.

Table S2. Contour length L, Kuhn length b, cylinder radius r, polydispersity factor PD, χ2, M_w, and radius of gyration R_g obtained from form factor fits using SasView to scattering curves measured for S0271 solutions at concentrations of 27.1 μM, 30.2 μM, and 45.7 μM 1h and 24h after sample preparation.

C /μM		L /nm	b /nm	r /nm	PD	χ^2	M_w /g mol^{-1}	R_g /nm
27.1	1h	724.3	129.9	5.0	0.4	0.97	$1.3 \cdot 10^8$	141.7
30.2	1h	6282.4	7.4	5.0	0.4	91.36	$9.9 \cdot 10^7$	99.5
45.7	1h	1020	116.9	5.0	0.4	2.26	$1.7 \cdot 10^7$	159.5
27.1	24h	1394.1	161.4	5.0	0.4	2.20	$3.7 \cdot 10^8$	219.1
30.2	24h	2044.7	95.4	5.0	0.4	1.23	$2.8 \cdot 10^8$	204.0
45.7	24h	1664.2	132.3	5.0	0.4	4.21	$9.1 \cdot 10^7$	216.8
27.1	1h	716.9	123.7	10	0.4	0.90	$1.2 \cdot 10^8$	137.6
30.2	1h	917.4	85.3	10	0.4	195.5	$9.8 \cdot 10^7$	129.2
45.7	1h	1031.4	113.3	10	0.4	2.31	$4.8\ 10^7$	157.9
27.1	24h	1220.3	165.1	10	0.4	2.43	$3.3 \cdot 10^8$	207.4
30.2	24h	1978.3	100.0	10	0.4	1.29	$2.9 \cdot 10^8$	205.5
45.7	24h	1627.4	135.9	10	0.4	4.34	$9.1 \cdot 10^7$	217.3

Form factor fits of the scattering data were performed with the flexible cylinder model included in the SasView software. The model uses, as parameters, the contour length of the cylinder L, the Kuhn length of the flexible cylinder b, the cross-section of the flexible cylinder r, and a scaling factor. A distribution of the contour length of the cylinders *f(x)* was considered via a Schulz distribution. The Schulz distribution is defined as

$$f(x) = \frac{1}{Norm}(z+1)^{z+1}\left(\frac{x}{\bar{x}}\right)^z \frac{\exp\left[-\frac{(z+1)x}{\bar{x}}\right]}{\bar{x}\Gamma(z+1)} \quad \text{(S1)}$$

where $\bar{x}$ is the number average contour length of the distribution, Norm is a normalization factor, which is determined during the numerical calculation, and z is a measure of the width of the distribution

$$z = \frac{1-PD^2}{PD^2} \quad \text{(S2)}$$

with PD as the extent of polydispersity given by

$$PD = \frac{\sigma}{\bar{x}} \quad \text{(S3)}$$

and σ as the root mean square deviation from $\bar{x}$.

The fitting procedure to the scattering curves was started by using the form factor of a monodisperse flexible cylinder in oder to adjust a scaling factor, the contour length L, the Kuhn length b and the radius of the cylinder r simultaneously to the scattering data recorded 1h and 24h after preparation. The fits resulted in values of r ~5 nm for the cylinder radius independent of the dye concentration. After having adjusted L, b and r to constant values, the distribution of the contour length was introduced in a second iteration. The resulting extent of polydispersities adopted values close to PD ~0.4 for all scattering curves, which can be transformed to z = 5.25 using eq S2. No deterioration of the fits was observed by keeping the dispersity of the contour length to a constant value of PD = 0.4.

In order to scrutinize the impact of aging on the thickness more carefully, we repeated the fits with r = 10 nm and r = 20 nm being fixed. Fits with a cylinder radius of r =

10 nm do not improve the fits significantly. The fits resulting from r = 20 nm (not shown) revealed higher fitting errors χ. Thus, an increase in the cylinder cross-section during aging can be excluded.

The squared radius of gyration was calculated using eq S4 [40]

$$R_g^2 = b^2 \left\{ \frac{u}{3} + 1 + \frac{2}{u} - \frac{2}{u^2} [1 - \exp(-u)] \right\} \tag{S4}$$

with

$$u = \frac{L}{b} \tag{S5}$$

$$R_g = \frac{\sum_{i=1}^{n} R_{g,i}^2 \cdot L_i^2 \cdot f(L)}{\sum_{i=1}^{n} L_i^2 \cdot f(L)} \tag{S6}$$

The weighted average molar mass was calculated according to

$$M_w = \frac{\sum_{i=1}^{n} M_i \cdot L_i \cdot f(L_i)}{\sum_{i=1}^{n} L_i \cdot f(L_i)} \tag{S7}$$

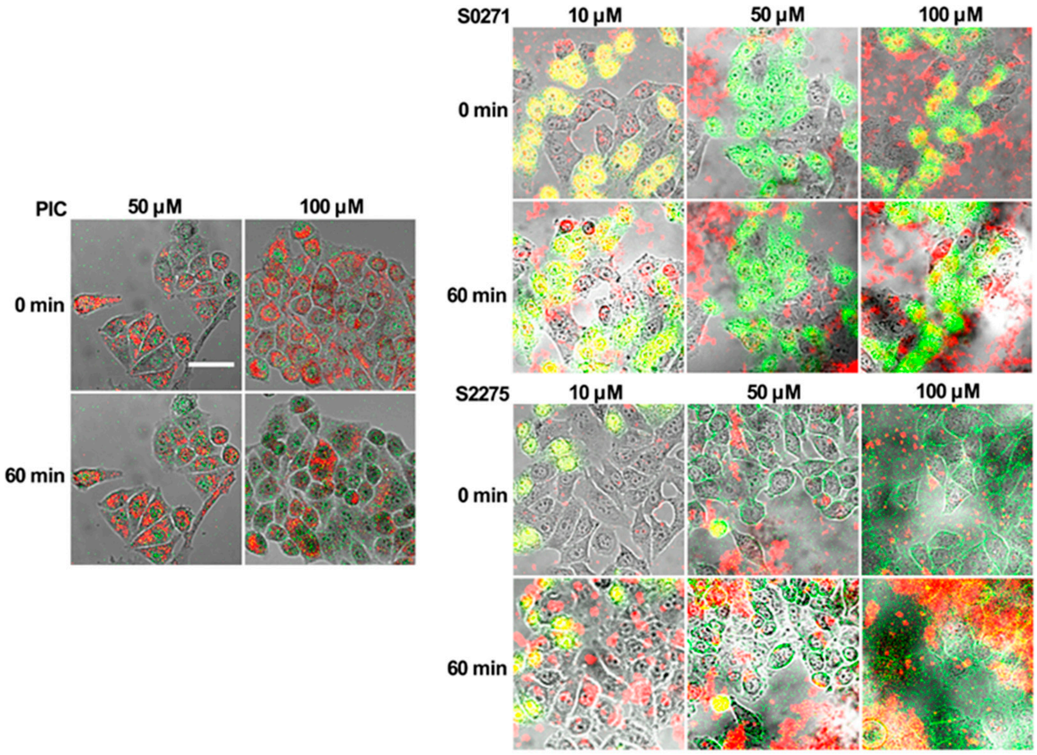

Figure S8. Exemplar HeLa cells treated with Leibovitz's medium supplemented with PIC (50 and 100 μM), S0271, and S2275 (10, 50, and 100 μM) at t = 0 min and 60 min after application. The monomer of each dye is displayed in green and the aggregate in red. The scale bar represents 50 μm.

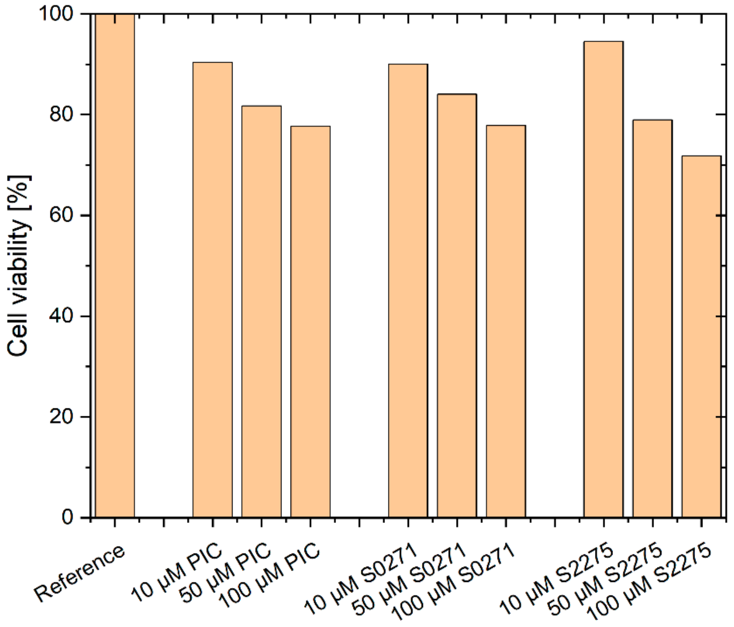

Figure S9. Visualization of the viability of the cells after 1 h of dye treatment.

HeLa cells were cultured in a growing medium (Dulbecco's Modified Eagle's Medium from Gibco, 10% fetal bovine serum from Sigma-Aldrich, 1% penicillin-streptomycin from Gibco) at 37°C and 10% CO_2 in T-25 culture flasks. At 90% confluence, cells were split into Fluorodishes of 10 mm diameter and incubated at 37°C and 10% CO_2 until next day's use. The cells were washed twice with 200 µl Dulbecco's Phosphate-Buffered saline (DPBS, from Gibco) and 200 µl dyestuff was added to the samples at concentrations of 1, 10, 50 and 100 µM in Leibovitz's solution. The samples were transferred into an incubation chamber (Okolab) attached to the microscope stage maintaining the samples at 37°C during the experiments. The cells were imaged after 0 and 60 min after dyestuff addition with a confocal microscope (Olympus IX83) using an excitation wavelength of 488 nm (PIC, S0271) and 561 nm (S2275). The detection wavelength was 510±20 nm for the monomer (S0271) and 570±30 nm for the aggregate (S0271) and 585±15 nm and 650±10 nm were used for S2275 as well as 530±20 nm and 575±10 nm for PIC, respectively. The data were evaluated via ImageJ 1.52a.
